# Using Pharmacologic Data to Plan Clinical Treatments for Patients with Peritoneal Surface Malignancy

**DOI:** 10.2174/157016309787581084

**Published:** 2009-03

**Authors:** Kurt Van der Speeten, Oswald Anthony Stuart, Paul H Sugarbaker

**Affiliations:** 1 Department of Surgical Oncology, Ziekenhuis Oost-Limburg, Genk, Belgium; 2 Washington Cancer Institute, Washington Hospital Center, Washington, DC, USA

**Keywords:** Intraperitoneal chemotherapy, 5-fluorouracil, doxorubicin, mitomycin C, peritoneal-plasma barrier, appendiceal cancer, colorectal cancer, peritoneal mesothelioma, ovarian cancer.

## Abstract

The surfaces of the abdomen and pelvis are an important anatomic site for the dissemination of gastrointestinal and gynecologic malignancy. This transcoelomic spread of cancer cells gives rise to peritoneal carcinomatosis which, without special treatments, is a fatal manifestation of these diseases. In order to control peritoneal carcinomatosis cytoreductive surgery to remove gross disease is combined with perioperative intraperitoneal and perioperative intravenous chemotherapy to eradicate microscopic residual disease. Chemotherapy agents are selected to be administered by the intraperitoneal or intravenous route based on their pharmacologic properties. A peritoneal-plasma barrier which retards the clearance of high molecular weight chemotherapy from the peritoneal cavity results in a large exposure of small cancer nodules on abdominal and pelvic surfaces. Tissue penetration is facilitated by moderate hyperthermia (41-42ºC) of the intraperitoneal chemotherapy solution. A constant dose of chemotherapy agent and volume of carrier solution based on body surface area allows prediction of systemic drug exposure and systemic toxicity. Timing of the chemotherapy as a planned part of the surgical procedure to maximize exposure of all peritoneal surfaces is crucial to success.

## INTRODUCTION

The peritoneal surface remains an important failure site for patients with gastrointestinal and gynecological malignancies. Besides the lymphatic and hematogenous routes of dissemination, transcoelomic spread of tumor cells is an acknowledged phenomenon ultimately giving rise to peritoneal carcinomatosis (PC). This intraperitoneal spread may occur before surgery as a direct consequence of full-thickness invasion of the involved organ by tumor and subsequently exfoliation of tumor cells in the peritoneal cavity. Also, intraperitoneal spread may be the result of surgical trauma that causes release of tumor cells from transected lymph and blood vessels and manipulation of the primary tumor. For example in a review of 2756 patients by Jayne *et al*., they reported the incidence of PC at the time of initial surgery to be 7.7% [1]. A review of colonic cancer patients who have recurrences suggests that peritoneal seeding occurred in 25-35% of patients [2].

## NATURAL HISTORY OF PERITONEAL CARCINOMATOSIS 

Nevertheless, little was done to clarify the impact of peritoneal seeding upon survival until the report of Chu and colleagues was published [3]. These investigators studied 100 patients with non-gynecologic malignancy that had biopsy-proven peritoneal carcinomatosis. The mean survival of 45 colorectal cancer patients was 8.5 months, of 20 pancreas cancer patients 2.4 months, and 6 gastric cancer patients 2.2 months. The presence or absence of ascites was an important poor prognostic variable in all of these patients.

In 2000 Sadeghi and coworkers reported on 370 patients with peritoneal carcinomatosis from non-gynecologic malignancies who were enrolled in a European prospective multicenter trial (Evolution of Peritoneal Carcinomatosis 1 [EVOCAPE 1]) [4]. These patients had the benefit of fluorouracil (5-FU)-based systemic chemotherapy, but the results were remarkably similar to those reported by Chu a decade earlier. The mean survival of 118 patients with carcinomatosis from colorectal cancer was 6.0 months, of 58 patients with pancreatic cancer 2.9 months, and of 125 patients with gastric cancer 6.5 months.

In 2002 Jayne and colleagues from Singapore used a database of 3019 colorectal cancer patients to identify 349 (13%) with peritoneal carcinomatosis [1]. Of special interest were the 125 patients (58%) who had synchronous primary colorectal cancer and peritoneal implants. The median survival of those patients was only 7 months. The authors reported that survival was adversely affected by the extent of the peritoneal carcinomatosis and the stage of the primary cancer.

## REVISED HYPOTHESIS REGARDING CARCINOMATOSIS

More recent chemotherapy based on the use of oxaliplatin, irinotecan and biological agents has improved survival in patients with metastatic colon cancer to 16-20 months [5-7]. Unfortunately, these recent trials do not provide data of results in patients with isolated PC and only address results in a collection of patients with metastatic colonic cancer at many different anatomic sites. In the past oncologists have assumed that PC is equal to distant metastases and as such regarded it as an incurable component of intraabdominal malignancy. PC has been regarded as beyond current treatment modalities. Over the last two decades a group of oncologists have worked using a revised hypothesis. Novel therapeutic approaches have emerged for patients with isolated peritoneal metastases of gastrointestinal cancer, ovarian cancer and primary peritoneal malignancies. These investigators all consider PC as a local-regional disease warranting local-regional chemotherapy (intraperitoneal) to treatment. *Spratt et al.,* reported for the first time in 1980 the use of heated triethylenethiophosphor-amide (thiotepa) in a patient with pseudomyxoma peritonei [8]. Koga *et al.* reported the use of intraperitoneal chemotherapy in 23 gastric cancer patients with PC [9]. Speyer in 1980 used normothermic intraperitoneal 5-FU and methotrexate in 16 patients with PC [10].

## ROLE FOR CYTOREDUCTIVE SURGERY

A second essential part of the modern management of PC is cytoreductive surgery with peritonectomy procedures. The underlying rationale of a combined approach was that on one hand an aggressive surgical approach combining visceral resections and peritonectomy procedures should address the macroscopic disease whereas perioperative intraperitoneal chemotherapy was aimed at residual microscopic disease [11]. The perioperative intraperitoneal chemotherapy includes Hyperthermic Intraperitoneal Chemotherapy (HIPEC) and/or Early Postoperative Intraperitoneal Chemotherapy (EPIC). More recent protocols advocate Bidirectional (simultaneous intraperitoneal and intravenous chemotherapy) Intraoperative Chemohyperthermia. This two-component approach of PC treatment requires that chemotherapy be used as a planned part of the surgical procedure. Critical to the success is proper timing of the chemotherapy in its relation to the surgery. This innovation of combined treatment of cytoreductive surgery plus perioperative intraperitoneal chemotherapy may be the paradigm shift responsible for recent successes versus prior failures in treating PC patients.

## RESULTS TO DATE IN TREATING CARCINOMATOSIS

Several phase II studies have explored the intraperitoneal route of drug delivery and show promising results in a variety of PC patients. In colorectal cancer with PC overall survival rates between 25% and 47% are reported [12-17]. These reports from many different institutions stand in strong contrast with historical control groups and patients treated with systemic chemotherapy where the mean and median overall survival was 6.0 months and 3.1 months [4]. Verwaal *et al.* in a phase III trial randomized patients with colorectal PC to palliative surgery followed by fluorouracil-leucovorin systemic chemotherapy versus maximal cytoreduction plus HIPEC with Mitomycin C and subsequent systemic chemotherapy [18]. This report showed a significant survival benefit for the HIPEC arm with a median survival of 22.3 months versus 12.6 months in the control group. Glehen *et al.* collected data retrospectively on 506 PC patients from 28 institutions treated with cytoreductive surgery and perioperative intraperitoneal chemotherapy [19]. He reported an overall median survival of 19.2 months. Patients in whom cytoreduction was complete had a median survival of 32.4 months versus 8.4 months in the patients with incomplete cytoreduction.

There is no doubt that the clinical evidence in the medical literature supporting the combined approach of cytoreductive surgery and perioperative intraperitoneal chemotherapy is growing [20-24]. What is lacking is clear understanding of the pharmacology of perioperative use of chemotherapy in the treatment of peritoneal surface malignancy. It is possible that increased safety and important treatment innovations may originate from analyzing the pharmacologic data. This review aims to clarify the pharmacokinetic and pharmacodynamic data currently available regarding the intraperitoneal delivery of cancer chemotherapy agents in patients with PC.

## THE PERITONEAL PLASMA BARRIER

The rationale of administering chemotherapeutic drugs into the peritoneal cavity is based on the relative transport barrier which is formed by the tissue surrounding the peritoneal space. The peritoneum is a complex three-dimensional organ covering the abdominopelvic organs and the abdominal wall. It contains a large potential space. The most elaborate description of the ultra structure of the peritoneum in man goes back to 1941 by Baron *et al.* [25]. The peritoneum consists of a monolayer of mesothelial cells supported by a basement membrane and five layers of connective tissue which account for a total thickness of 90 µm. The connective tissue layers include interstitial cells and a matrix of collagen, hyaluron, and proteoglycans. The cellular component consists of pericytes, parenchymal cells and blood capillaries. The complex is often referred to as the peritoneal membrane. This description is a working model derived from research regarding the peritoneum as a dialysis membrane.

The accepted function of the peritoneum is twofold. First, it reduces friction between intraabdominal organs and the abdominal wall by producing a lubricant solution made of glycosaminoglycans and phospholipids [26]. Secondly, it is of major importance together with lymphoid aggregates dispersed on the visceral and parietal peritoneum in the host defense against intraabdominal infections. A third suggested function of the peritoneum in malignancy may be its role as a first line of defense against peritoneal carcinomatosis [27]. Any disruption in the peritoneal lining facilitates the adhesion-invasion cascade of tumor cells, resulting in the development of peritoneal tumor nodules on the abdominal or pelvic surface [27, 28].

Contrary to intuitive thinking the elimination of the mesothelial lining as performed during peritonectomy procedures does not significantly alter the pharmacokinetic properties of the peritoneum in the transport of chemotherapeutic agents from the peritoneal cavity to the plasma compartment. Flessner *et al*. demonstrated in a rodent model that neither removal of the stagnant fluid layer on the mesothelium nor removal of the mesothelial lining influenced the mass transfer coefficient (MTC) over the barrier [29]. Indirect evidence supporting this hypothesis in humans can be derived from the fact that the extent of the peritonectomy in PC patients does little to alter the intraperitoneal chemotherapy pharmacokinetics of Mitomycin C or 5-fluorouracil [30, 31]. Basic research rather demonstrates that not only the mesothelial lining but also the blood capillary wall and the surrounding interstitial matrix are the principal barrier for clearance of molecules from the abdominopelvic space [32].

**Fig. (1):**
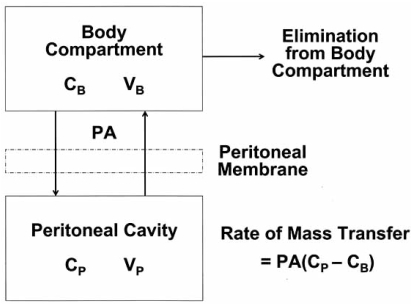
**Traditional two-compartment model of peritoneal transport in which transfer of a drug from the peritoneal cavity to the blood occurs across the “peritoneal membrane”. The permeability-area product (*PA*) governs this transfer and can be calculated by measuring the rate of drug disappearance from the cavity and dividing by the overall concentration difference between the peritoneal cavity and the blood (or plasma). *C_B_* = the free drug concentration in the blood (or plasma); *V_B_* = volume of distribution of the drug in the body; *C_p_* = the free drug concentration in the peritoneal fluid; *V_p_* = volume of the peritoneal cavity.** (Dedrick R.L., Flessner M.F.:* Pharmacokinetic problems in peritoneal drug administration: Tissue penetration and surface exposure.* J Natl Cancer Inst 1997; 89(7): 480-87).

Most basic research concerning the pharmacokinetic properties of the peritoneum is derived from the peritoneal dialysis literature [33]. A simplified mathematical diffusion model considers the plasma to be a single compartment separated by an effective membrane from another single compartment, the peritoneal cavity (Fig. **1**). This results in the following equation:

Rate of mass transfer = PA (C_P_ - C_B_) where PA = permeability area, C_P_ = concentration in peritoneal cavity and C_B_ = concentration in the blood.

Although this offers a simple conceptual model of transport and states the importance of the effective exposure area, it only offers quantitative predictability once PA is empirically determined for each drug. It also does not offer insight into the actual tissue penetration at the level of the peritoneal membrane. Neither does it predict penetration of chemotherapy into the tumor nodules which is the single most important factor determining response to cancer treatment. 

## PHARMACOKINETIC RATIONALE OF PERIOPERATIVE INTRAPERITONEAL CANCER CHEMOTHERAPY

Intraperitoneal administration of chemotherapeutic agents gives high response rates in PC patients because the peritoneal plasma barrier provides dose-intensive therapy. Based on peritoneal dialysis research, Dedrick *et al.* in 1978 concluded that the peritoneal permeability of a number of hydrophilic anticancer drugs may be considerably less than the plasma clearance of that same drug. This results in a significantly higher concentration in the peritoneal cavity as compared to the plasma after intraperitoneal administration [34]. This concentration difference offers the opportunity of exposing the residual tumor cells after cytoreduction to high doses of chemotherapeutic agents with reduced systemic concentrations and lower systemic toxicity. This advantage is expressed by the Area Under the Curve (AUC) ratios of intraperitoneal versus plasma exposure. Table **1** presents molecular weight and AUC IP/IV for drugs in clinical or experimental use in PC patients [35].

An important consideration is that high intraperitoneal concentration or AUC IP/IV does not automatically confer a greater efficacy. Even with greatly elevated intraperitoneal cancer chemotherapy concentrations, there may be limited penetration of the chemotherapeutic agent into the peritoneal tumor target. The ideal drug for intraperitoneal chemotherapy has a high peritoneal tissue concentration as a result of direct intraperitoneal administration and a high penetration into the cancer nodule. This should occur along with slow diffusion through the capillary endothelium deep in the subperitoneal space of the cancer chemotherapy solution. Low systemic concentrations and reduced systemic toxicity are maintained by rapid metabolism and excretion of drug within the body compartment.

The marked increase in exposure of peritoneal surfaces to chemotherapy solution as compared to plasma is illustrated in Fig.(**2**). The chemotherapy agent, paclitaxel, has a high molecular weight (853.9 Daltons) and is slow to cross the peritoneal cavity to plasma barrier. The naked molecule of paclitaxel is highly lipophilic. It is unique in cancer chemotherapy in that the intravascular or intraperitoneal administration of the drug requires it to be suspended in a detergent that maintains the drug in solution. The detergent molecules surround the paclitaxel molecule giving it additional size and a hydrophilic character. This large molecular size and hydrophilic behavior of the complex molecule result in slow passage across the peritoneal to plasma barrier. The AUC ratio for paclitaxel is 1000 (Table **1**).

## PHARMACOKINETIC RATIONALE OF PERIOPERATIVE INTRAVENOUS CANCER CHEMOTHERAPY

New pharmacologic data suggests altered parmacodynamics of intravenously administered cancer chemotherapy drugs when used intraoperatively during a HIPEC procedure. Fig. (**3**) shows 5-fluorouracil concentrations in the plasma, peritoneal fluid and tumor nodules after intravenous administration at the beginning of the HIPEC procedure. The intravenous fluorouracil is rapidly transported from the plasma compartment to the expanded peritoneal cavity. There it is retained in the artificial ascites created by the HIPEC for a substantial amount of time before a reduced rate of reabsorption into the systemic compartment occurs. Our initial data suggest that the artificial ascites created by the HIPEC may provide a reservoir for the intravenous administered drug. The intraoperative administration of intravenous cancer chemotherapy drugs may offer a pharmacologic advantage in killing residual tumor cells after cytoreduction. Timing of intravenous cancer chemotherapy (intraoperative versus pre- or postoperative) emerges as a new variable which may affect efficacy of cancer chemotherapy drugs in treating patients with a peritoneal surface malignancy.

## TISSUE DISTRIBUTION AND PENETRATION DEPTH

The simplified two-compartment model described above may not provide an adequate theoretical model for penetration of the intraoperatively administered (either intravenous or intraperitoneal) chemotherapy into the peritoneal wall and into the tumor nodules. Dedrick *et al*. proposed a mathematical model seen in Fig. (**4**) addressing the tissue penetration of low-molecular weight molecules [36, 37]. The drug diffuses from its peritoneal concentration, C_p_, to its blood concentration, C_B_, along an exponential concentration gradient over the peritoneum and preperitoneal tissues. The extracellular ‘deep’ concentration, C_e_, can then be calculated according to the formula:

$$C_{e}=C_{B}+\left(C_{p}-C_{B}\right)\text{exp}\left[-{\left(k/D\right)}^{1/2}x\right]$$

In this formula k (min^-1^) is the rate constant for removal of the active drug from the tissue. Movement through the tissue is characterized by the diffusivity, D (cm^2^/min) and x is the distance from the serosal surface (cm). This model implies that there is an exponential concentration decrease of the drug from abdominopelvic cavity across the membrane to the plasma compartment. Consequently, the depth of penetration of an effective chemotherapy concentration is very limited and is in the order of 1 to 2 mm [38, 39]. Ozols *et al.* confirmed adriamycin penetrating only 4-6 cell layers of tumor on the diaphragm in a rodent model [40]. In all likelihood there is a variable penetration for each drug and type of tumor.

This has important consequences for implementing perioperative chemotherapy in PC patients. The cytoreduction needs to eliminate all tumor deposits greater than 1-2 mm for the subsequent intraperitoneal chemotherapy to be effective. Clinical data to support this pharmacologic prediction is strong; in univariate and multivariate analysis the complete cytoreduction (cancer nodules ≤ 2.5 mm) is the single most important prognostic factor [15-19]. 

In order to describe the pharmacology of both intravenous and intraperitoneal chemotherapy used after cytoreduction to treat PC a revised theoretical model is needed. Fig. (**5**) shows a three-compartment model constructed of a body compartment, intermediate compartment (peritoneal and preperitoneal tissues with tumor nodules) and peritoneal fluid. The movement of chemotherapy molecules from blood to peritoneal fluid through the intermediate compartment should be rapid because of the extensive arterial and portal venous blood delivery to the intermediate tissues. The movement of chemotherapy molecules from peritoneal fluid to portal blood would be less rapid.

The number of variations in intraperitoneal chemotherapy treatment protocols is extensive. All these variations reflect attempts to improve diffusivity D, decrease the rate constant k, permeability P or effective membrane area A. A non-exhaustive list of factors influencing these values and thus response of the peritoneal metastases to the drug is listed in Table **2**. Some of these will be explored in greater detail.

## TEMPERATURE

Adding hyperthermia to intraperitoneal chemotherapy may increase the tumor response to cancer chemotherapy drugs by several mechanisms. First, heat alone has a direct anti-tumor effect. Hyperthermia above 41°C induces selective cytotoxicity of malignant cells. Several mechanisms have been proposed: impaired DNA repair, protein denaturation and inhibition of oxidative metabolism in the microenvironment of malignant cells leading to increased acidity, lysosomal activation and increased cell death [41, 42]. Cells are known to react to protein denaturation by up regulation of heat shock proteins. This induces the development of thermal tolerance in tumor cells [43]. Thermal tolerance may cause the clinical importance of this first mechanism to be limited. Second, the cytotoxic effects of some chemotherapeutic agents are augmented by applying mild hyperthermia. Such augmented effects were postulated for doxorubicin [44], platinum complexes [45, 46], mitomycin C [46], melphalan [47], docetaxel, irinotecan and gemcitabine [48]. Third, this increased response may be the consequence of the altered penetration depth of the chemotherapeutic agent [49, 50].

Jacquet and colleagues studied the changes in the penetration of intraperitoneal chemotherapy that are induced by moderate hyperthermia. Large and statistically significant increases in the amount of doxorubicin within tissues were demonstrated. The tissues in direct contact with the intraperitoneal chemotherapy were most affected. The concentration of doxorubicin in small bowel was doubled using intraperitoneal doxorubicin plus heat; a similar finding occurred for omental tissue and bladder tissue. Tissues at a distance such as heart muscle, did not show an increased concentration with heat. The heat utilized by Jacquet and colleagues within the peritoneal cavity was 41.5 to 42 ºC in this rat model [50].

## PRESSURE

Dedrick *et al.* postulated that the penetration distance is equal to the square root of the ratio of the tissue diffusivity and the rate constant for drug removal from the tissue (D/k)^1/2^. Unpublished observations by Flessner in a rat model showed a doubling of the extracellular space in the anterior abdominal wall of rats when the pressure of intra-abdominal peritoneal dialysis solution was raised from 0 to 4 cm H_2_O [29]. An increased effective diffusivity was postulated.

Animal experiments [51, 52] confirmed the increased intratumoral accumulation and antitumor effect of intraperitoneal doxorubicin and cisplatin when the intraabdominal pressure was raised. Increased intraabdominal pressure is thought to generate a convective flux that forces the drug from the peritoneal cavity into the subperitoneal tissue. At the same time intraabdominal pressure may counteract the hydraulic capillary pressure and slow the outflow of the drug to the body compartment. Measurement of local cisplatin concentrations along the radii of peritoneal tumor nodules showed platinum penetration far beyond the 1 mm limit advocated by Los *et al*. [39]. The clinical limit of usable intraabdominal pressure enhancement is dictated by respiratory and hemodynamic tolerance. Clinical applications of HIPEC in intraabdominal pressure settings so far has been limited to palliating debilitating malignant ascites with laparoscopic HIPEC at 10-15 mm Hg [53, 54].

## CARRIER SOLUTION

A variety of carrier solutions have been applied in different treatment protocols. Hypotonic, isotonic and hypertonic solutions were explored with both low and high molecular weight chemotherapy molecules. The ideal carrier solution should enhance the exposure of the peritoneal surface and residual tumor cells to the chemotherapeutic agent. This is especially important in the setting of EPIC where maintenance of a high dwell volume of perfusate over a prolonged time period improves the distribution of the drug and the effectiveness of the treatment. Chemotherapy solutions of isotonic and hypertonic salt, dextrose, hetastarch or icodextrin solutions have been explored. In an EPIC setting a high molecular weight solution that maintains an artificial ascites showed a higher drug availability because the total drug exposure depends not only on the active drug but also on the carrier solution that maintains the peritoneal fluid volume [55-57]. 

In a HIPEC setting with a relatively short dwell time, one could theoretically expect a pharmacodynamic advantage of a hypotonic carrier through the mechanism of increased tissue and tumor absorption. Contrary to experimental studies supporting this hypothesis, Elias *et al.* [58] showed in humans no increase in tumor penetration. A concomitant high incidence (50%) of postoperative peritoneal bleeding and severe thrombocytopenia has contraindicated the further clinical use of hypotonic carriers.

## VOLUME OF CHEMOTHERAPY SOLUTION

Since peritoneal metastases and free floating tumor cells can be present anywhere on the peritoneal surface, the entire surface of the abdominal and pelvic cavity is the target. Substantial differences in body composition of patients and differences in the actual HIPEC technique (open versus closed abdomen) will result in a wide variety of perfusate volumes. In current practice the volume of the perfusate is chosen quite arbitrary. Following the above stated (equation) concerning mass transfer over the peritoneal-plasma membrane increasing the solution contact area A improves the mass transfer. Keshaviah demonstrated a linear rise in mass transfer in 10 patients who were dialyzed with different volumes ranging from 0.5 up to 3 liters [59]. Elias first published the importance of volume of chemotherapy in determining systemic exposure to the drug [60]. Sugarbaker *et al*. carried out a clinical investigation where 2 versus 4 versus 6 liters of chemotherapy solution were administered. The dose of chemotherapy solution in these studies was constant. They showed that a more dilute intraperitoneal chemotherapy concentration retarded the clearance of chemotherapy and lead to a lesser systemic toxicity [61]. Also, it must be assumed that the less concentrated chemotherapy would, by the diffusion model, penetrate less into the cancer nodules and into normal tissues. These authors determined it necessary to regulate not only the chemotherapy dose but also the volume of chemotherapy solution by the patient’s body surface area.

A consistent drug dose and chemotherapy solution volume may be the optimal method to predict a maximal treatment in the abdomen with a predictable bone marrow toxicity. Sugarbaker and colleagues suggested that variable volume is a dangerous practice with unpredictable systemic toxicities [61]. If chemotherapy solution is administered until the abdomen is full the contact area will increase. If the contact area is variable the total absorption of drug cannot be predicted. 

## VASOACTIVE AGENTS

The literature concerning the effects of vasoactive substances in regulating peritoneal blood flow and tumor blood flow is extensive. These agents may contribute to a delayed clearance from the peritoneal cavity because the blood flow in the peritoneal and subperitoneal vascular network will control, in large part, the movement of molecules across the peritoneal and subperitoneal tissues. General statements regarding the effects of vasoactive agents are confusing and sometimes contradictory due to the variety of experimental systems, complex interactions of local-regional and systemic effects of vasopressive agents, and large differences between the neovasculature of tumor nodules and normal capillaries. Both intravenous and intraperitoneal administration of vasoactive molecules in combination with chemotherapeutic drugs has been explored [36, 62, 63]. A preclinical study of the use of an intraperitoneal epinephrine plus intraperitoneal cisplatin in a rat model with PC showed a direct correlation between the intraperitoneal epinephrine concentration and cisplatin accumulation in rat peritoneal tumor nodules [64]. Recently, Molucon-Chabrot and colleagues for the first time demonstrated the safe use of intraperitoneal epinephrine with intraperitoneal cisplatin in 18 patients with advanced peritoneal carcinomatosis [65]. Tumor responses were obtained in some patients resistant to intravenous platinum compounds. Lidner *et al.,* reported that concurrent intravenous administration of vasopressin can increase the pharmacokinetic advantage of intraperitoneal administered carboplatin and etoposide but not 5-FU [66]. Further studies on the use of vasoactive agents to improve cancer chemotherapy responses in PC are needed.

## DOSE OF INTRAPERITONEAL CANCER CHEMOTHERAPY

Many different chemotherapy agents, drug concentrations, drug doses, and drugs schedules have evolved at many institutions for perioperative cancer chemotherapy. Most authors use a drug dose based on calculated body surface area (mg/m^2^) although Rubin *et al*., demonstrated that there is an imperfect correlation between actual peritoneal surface area and calculated body surface area [67]. The same study suggests sex differences in peritoneal surface areas which in turn might affect the absorption characteristics. The female has a larger peritoneal surface in proportion to body size than the male by approximately 10%. Estimates of the functional peritoneal surface area by applying stereologic methods to CT scans have been attempted [68]. 

Body surface area is an accurate predictor of drug metabolism and in this regard is useful for estimating systemic drug toxicity. As discussed by Sugarbaker *et al*., the accuracy of this prediction is increased if the volume of chemotherapy solution is also determined by the body surface area [61]. With a constant total dose of chemotherapy and chemotherapy solution the bone marrow exposure to cytotoxic drugs can be most accurately predicted. If these predictions are not available, the danger of overdosing some patients and underdosing others will occur. 

A significant number of institutions using a closed method for intraoperative hyperthermic chemotherapy calculated the dose of cancer chemotherapy per liter by body surface area. The total amount of cancer chemotherapy is mixed in a large volume of carrier solution (usually six liters) that is placed in a reservoir. For example, Deraco *et al*. have used doxorubicin 15.25 mg/m^2^/l and cisplatin 43 mg/m^2^/l with the total volume of 6 liters [69, 70]. Gilly and coworkers have used mitomycin C 0.5 mg/kg and cisplatin 0.7 mg/kg in a total volume of 4 to 6 liters [71, 72]. In this method the amount of chemotherapy solution in contact with the peritoneal surface is determined by multiple variables - the amount of distention (between 2 and 6 liters) of the abdominal cavity induced by the chemotherapy solution, the patient sex, the amount of ascites present preoperatively and the extent of the visceral resection. 

In summary, this system that allows a variable amount of chemotherapy solution may result in a less accurate prediction of plasma AUC. The total volume of intraperitoneal chemotherapy can vary widely betweens individuals. Increases in the volume of intraperitoneal chemotherapy solution cause an increase in diffusion surface and an increase in the amount of drug from peritoneal space to plasma.

## DISTRIBUTION OF INTRAPERITONEAL CHEMOTHERAPY

**Fig. (6):**
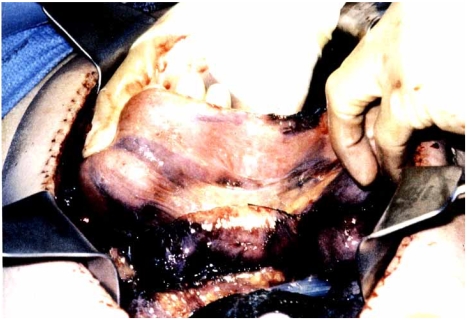
**Spatial distribution of intraperitoneal methylene blue using the closed abdomen technique.** Although the subcutaneous tissues were uniformly stained, the small bowel loops showed variable staining caused by adherence of adjacent bowel loops.

A wide variety of open and closed intraperitoneal chemotherapy methods of administration have been described [73-77]. The closed abdomen techniques have the theoretical advantage of less heat loss during perfusion. Also, a reduction in environmental risk for the operative team has been suggested. Open abdomen techniques will provide a better spatial distribution of the chemotherapy solution over the entire peritoneal surface. Also, a more uniform heat distribution is expected if the chemotherapy solution is manually stirred throughout the HIPEC. However, to date no study has been able to detect any occupational risk for the staff [78-80]. Elias *et al*. performed a prospective phase I-II study comparing seven HIPEC perfusion techniques [76]. Judgments regarding adequate spatial distribution were made by measurement of temperature at multiple sites. Using the closed system, temperature varied greatly throughout the abdomen and pelvis. The temperature became more uniform with an open abdomen and manual distribution. Spatial diffusion, studied by adding methylene blue to the perfusate, was incomplete in the closed abdomen techniques (Fig. **6**). However, these differences in the delivery of HIPEC may be more theoretical than real. In studies to date only the extent of PC, the absence of extraperitoneal disease and the completeness of cytoreduction correlated with survival. A comparison of data from centers performing either closed or open abdomen techniques suggest that the chemotherapy effects by open or closed methods may be similar. Long follow-up will be required to make an accurate comparison.

## TIMING OF CANCER CHEMOTHERAPY IN RELATION TO TIMING OF THE SURGICAL INTERVENTION

### Neoadjuvant Bidirectional Chemotherapy

Considering the clinical application of chemotherapy in PC patients, one can intervene at four possible points in the timeline. First, *neoadjuvant bidirectional chemotherapy uses both the intraperitoneal and intravenous routes of chemotherapy administration.* It has been explored as an option to reduce the extent of small PC nodules. Theoretically, it may facilitate definitive cytoreductive surgery after initial exploratory laparoscopy or laparotomy. This approach was acronymed as Neoadjuvant Intraperitoneal and Systemic chemotherapy (NIPS) [81]. Radiologic and clinical responses have been reported by several groups [81-83]. Although this strategy may reduce the tumor load to be addressed by cytoreductive surgery, it has several disadvantages. Adhesions from prior surgical interventions may interfere with adequate intraperitoneal drug distribution. Also, complete responses are very unusual so that further cytoreduction-chemotherapy is definitely necessary if the approach is curative. Neoadjuvant intraperitoneal chemotherapy has been reported to add to morbidity and mortality of further surgical treatment [84]. Extensive fibrosis as a response to chemotherapy may occur and make judgments concerning the extent of peritoneal carcinomatosis difficult – even impossible to assess.

### Intraoperative Intraperitoneal Chemotherapy

*Intraoperative intraperitoneal chemotherapy* has been the most widely explored modality with consistent clinical improved outcomes in many phase II trials and several phase III trials [12-24]. 

### Early Postoperative Intraperitoneal Chemotherapy

*Early postoperative intraperitoneal chemotherapy* has some conceptual advantages. It is administered after cytoreductive surgery at the time of minimal residual tumor burden. Also, intraperitoneal treatments initiated before wound healing occurs, can minimize non-uniform drug distribution and eliminate residual cancer cell entrapment in postoperative fibrin deposits. Proper selection of chemotherapy agents based on pharmacologic principles suggests the use of cell-cycle specific drugs such as 5-fluorouracil and the taxanes. Most EPIC regimens are administered postoperatively day 1 to 5 or day 1 to 4 through an inflow catheter and outflow drains placed at the time of cytoreductive surgery. EPIC can be applied with or without HIPEC [85]. 

*Long-term combined intraperitoneal and systemic chemotherapy*. Markman *et al*. Alberts *et al*. and Armstrong plus coworkers demonstrated in a phase III trials that intravenous plus intraperitoneal chemotherapy improves survival in patients with optimally debulked stage III ovarian cancer as compared to intravenous chemotherapy alone [86-88]. This approach may be used as ‘chemotherapeutic bridging’ between incomplete initial surgery and definitive cytoreduction or second look surgery. This type of chemotherapy is an adjuvant and not a perioperative use of chemotherapy. Failure analysis reported for cytoreductive surgery plus perioperative chemotherapy determined recurrent cancer most frequently occurs within the abdominal and pelvic cavity [89, 90]. Although systemic metastases do occur, treatment failures rarely occur in liver, lungs or other systemic sites. In order to optimize the treatment of patients with PC it is likely that the greatest benefit will occur from a combination of these four treatment strategies.

## DURATION

A wide variety of durations for HIPEC have been reported ranging from 30 to 120 minutes. The duration is not arbitrary and selection should proceed according to the pharmacologic clearance of the chemotherapeutic drug. 

## MACROMOLECULAR VEHICLES

In recent years an increased interest in macromolecular vehicles and other modulations of chemotherapeutic agents as a means of exploiting the regional dose intensity has emerged. The results of this research are conflicting. Contrary to intuitive thinking macromolecules may penetrate more deeply in the subperitoneal space despite their lower diffusivities. The nature of the capillary permeability probably is the major factor responsible for this higher concentration in the subperitoneal space together with an increased role of convection [91]. One should be cautious to conclude that this increased penetration into the subperitoneal space results in increased drug absorption into tumor nodules. One should not assume that the neovascularity of tumor nodules has the same selectivity for macromolecules as normal capillaries [92]. A second obstacle to cancer chemotherapy penetration into tumor nodules concerns the interstitial pressures in tumor nodules. It is significantly higher than that of the surrounding tissue space [93]. Convection may reduce tumor penetration by macromolecules. 

## INDIVIDUAL DRUG SENSITIVITY OF TUMORS WITHOUT AND WITH HYPERTHERMIA

The selection of chemotherapeutic agents used in perioperative chemotherapy protocols has been based on research in chemotherapeutic responses in systemic administration, on pharmacodynamic and pharmacokinetic properties of the drug in intraperitoneal administration, increased cytotoxicity with hyperthermia and synergy between chemotherapeutic agents. There is solid evidence supporting a tumor-specific heterogeneous activity of cytotoxic drugs in cell cultures of different tumors [94, 95].

Mahteme *et al*. recently stated the same heterogeneous cytotoxic response of cytotoxic drugs in PC samples in a variety of tumors [96]. The clinical implication of these data justifies further research towards an individualized selection of drugs in PC patients. However, it should be acknowledged that as yet there is no prospective data supporting an improved clinical outcome from drug selection based on in-vitro drug sensitivity testing.

## BIDIRECTIONAL INTRAOPERATIVE CHEMOTHERAPY

The three compartment model described above for peritoneal transport predicts transport by diffusion from the peritoneal compartment through a peritoneal and preperitoneal tissue layer to the plasma. Also, drugs move from the plasma compartment through the preperitoneal tissue layer to the peritoneal compartment. By combining intraoperative intravenous and intraoperative intraperitoneal cancer chemotherapy a bidirectional diffusion gradient is created through the intermediate tissue layer which contains the cancer nodules. This offers opportunities for optimizing cancer chemotherapy delivery to the target peritoneal tumor nodules. Elias and coworkers were the first to utilize this approach [60]. Further pharmacologic studies are needed to clarify the most efficient method of administration (continuous versus bolus versus repeated bolus), doses and choice of cancer chemotherapy drugs for this bidirectional approach.

## CONCLUSION AND FUTURE DIRECTIONS

The administration of perioperative chemotherapy in patients with peritoneal carcinomatosis should be governed by pharmacologic principles. Patients who have minimal residual disease as a result of cytoreductive surgery are candidates for perioperative chemotherapy by the intraperitoneal and intravenous route. Hyperthermia of the intraperitoneal chemotherapy solution will increase the cytotoxicity of the drug within the peritoneal cavity. Heating of the peritoneal and preperitoneal tissues will maximize the systemic chemotherapy effects on carcinomatosis, a phenomenon known as heat targeting. Perioperative chemotherapy has become an important part of cancer treatment and should become a standard modality for prevention and treatment of a wide variety of cancers that involve the peritoneal surfaces.

In November of 2006 a consensus meeting was held in Milan to address current standard of practice in the clinical application of cytoreductive surgery and hyperthermic intraperitoneal chemotherapy in the management of patients with cancer [97]. The applications, both for the prevention of carcinomatosis and the management of carcinomatosis, were explored and suggestions regarding the current standard of practice offered by the consensus group using the Delphi method. It was clear that for two diseases combined treatment is now considered the standard of care. For mucinous appendiceal neoplasms with peritoneal dissemination a curative treatment is available in three-quarters of the patients who have a minimally aggressive cancer. For those patients who had a complete cytoreduction, half are alive and well at 10 years. There are no 10 year survivors in the absence of these treatments. Similarly, in patients with peritoneal mesothelioma a prior standard of care using systemic chemotherapy offered approximately a 1 year median survival [20]. Using cytoreductive surgery and perioperative intraperitoneal chemotherapy this median survival has been extended to 5 years. Also, patients with carcinomatosis from colon cancer who have a small volume of cancer disseminated to the peritoneal surfaces and who are able to undergo complete cytoreduction have a median survival of approximately 30 months and a 5-year survival of approximately 40%. Again, this approach is considered a standard of care with small volume carcinomatosis from colon cancer. The national health care systems of the Netherlands and France have approved this approach for colon carcinomatosis. In other countries in Europe, approval comes on a case-by-case basis. 

In two other major diseases early results of treatment as studied in meta-analyses and systematic reviews suggest a role for this combined treatment in ovarian cancer [21]. Treatment at the time of diagnosis was suggested by the consensus group as the most likely to favorably affect survival. Currently, this combined treatment is most frequently used as a salvage treatment after systemic chemotherapy has failed. Even in this setting the results strongly suggest prolonged survival. With gastric cancer the meta-analysis strongly suggests an adjuvant role for perioperative intraperitoneal chemotherapy in preventing local-regional failure of this disease after gastrectomy. Although this comprehensive approach to the treatment of primary gastric cancer has only been widely employed in Korea, it has advocates worldwide. Also, the best palliation of gastric cancer with peritoneal seeding may come from neoadjuvant intraperitoneal and systemic chemotherapy in patients with peritoneal carcinomatosis from this disease. In addition, with this approach a small percentage of patients (approximately 20% of those treated) may come to a complete cytoreduction when gastrectomy and peritonectomy are utilized. Applications of this method of treatment are currently under investigation for pleural mesothelioma, endometrial cancer, and retroperitoneal and visceral sarcoma.

## Figures and Tables

**Fig. (2):**
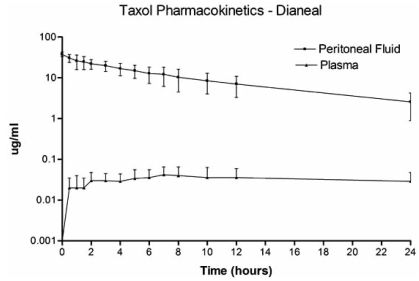
Pharmacokinetic study of concentration versus time for intraperitoneal paclitaxel. The chemotherapy agent at 30 mg/m^2^ was instilled directly into the peritoneal cavity as rapidly as possible in a 1.5% dextrose peritoneal dialysis solution. The concentration of paclitaxel was determined in peritoneal fluid and in plasma for 24 hours.

**Fig. (3):**
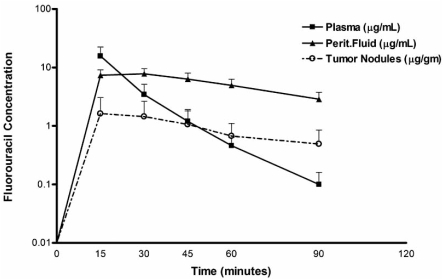
**Pharmacodynamics during hyperthermic intraperitoneal chemotherapy (HIPEC) after intravenous administration of 400 mg/m^2^ of 5-fluorouracil given simultaneously with intraperitoneal chemotherapy in 3 liters of chemotherapy solution.** (Van der Speetan K., Stuart O.A., Mahtsme H., Sugarbaker P.H.: *Pharmacology of perioperative 5-fluorouracil*, in press, 2009).

**Fig. (4):**
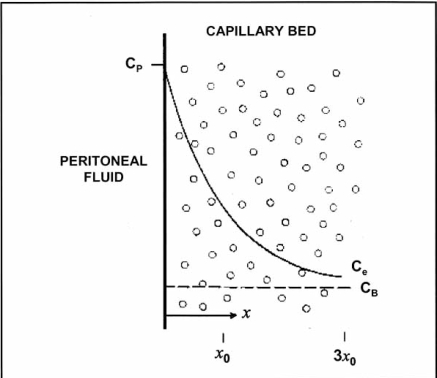
**Conceptual diagram of tissue adjacent to the peritoneal cavity. *C_p_* = the free drug concentration in the peritoneal fluid; *C_B_* = the free drug concentration in the blood (or plasma). Solid line shows the exponential decrease in the free tissue interstitial concentration, *C_e_*, as the drug diffuses down the concentration gradient and is removed by loss to the blood perfusing the tissue. Also shown are the characteristic diffusion length, x_0_, at which the concentration difference between the tissue and the blood has decreased to 37% of its maximum value, and 3x_0_, at which the difference has decreased to 5% of its maximum value.** (Dedrick R.L., Flessner M.F.: *Pharmacokinetic problems in peritoneal drug administration: Tissue penetration and surface exposure.* J Natl Cancer Inst 1997; 89(7): 480-87).

**Fig. (5):**
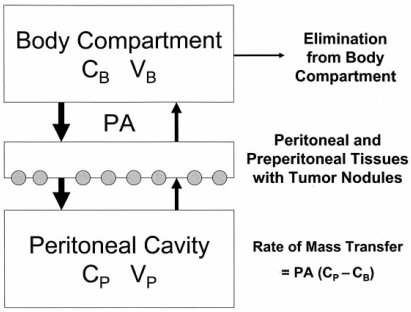
**Three-compartment model of peritoneal transport in which transfer of a drug from the peritoneal cavity to the blood occurs across the peritoneal membrane and preperitoneal tissues. In these tissues the peritoneal surface cancer nodules are located. The permeability-area product (*PA*) governs this transfer and can be calculated by measuring the rate of drug disappearance from the cavity and dividing by the overall concentration difference between the peritoneal cavity and the blood (B). *C_B_* = the free drug concentration in the blood (or plasma); *V_B_* = volume of distribution of the drug in the body; *C_P_* = the free drug concentration in the peritoneal fluid; *V_P_* = volume of the peritoneal cavity.** (Modified from Dedrick R.L., Flessner M.F.: *Pharmacokinetic problems in peritoneal drug administration: Tissue penetration and surface exposure.* J Natl Cancer Inst 1997; 89(7): 480-87).

**Table 1.** Molecular Weight and Area Under the Curve Ratios of Intraperitoneal Exposure to Systemic Exposure of Chemotherapeutic Agents Used to Treat Peritoneal Carcinomatosis

Drug	Molecular Weight (Daltons)	Area Under the Curve Ratio
5-Fluorouracil	130.08	250
Carboplatin	371.25	10
Cisplatin	300.1	7.8
Docetaxel	861.9	552
Doxorubicin	579.99	230
Etoposide	588.58	65
Floxuridine	246.2	75
Gemcitabine	299.5	500
Irinotecan	677.19	N/A
Melphalan	305.2	93
Mitomycin C	334.3	23.5
Mitoxantrone	517.41	115-255
Oxaliplatin	397.3	16
Paclitaxel	853.9	1000
Pemetrexed	597.49	40.8

**Table 2.** Variables Influencing the Response of Peritoneal Carcinomatosis to Perioperative Chemotherapy

Temperature
Dose of intraperitoneal chemotherapy
Distribution of chemotherapy solution and heat
Timing of chemotherapy in relation to the timing of the surgical intervention
Type of carrier solution
Pressure
Volume of carrier solution
Duration of instillation
Vasoactive agents
Macromolecular vehicles
Drug sensitivity of the tumor
Size of residual tumor nodules
